# Whole genome sequencing reveals a specific microbiota in subglottic stenosis *C. acnes* may contribute to inflammation

**DOI:** 10.1007/s00405-026-10630-0

**Published:** 2026-09-17

**Authors:** Anders Erlandsson, Stina Chorell, Anne Tuiskunen Bäck, Linda Köhn, Michael Haney, Katarina Olofsson

**Affiliations:** 1 Department of Clinical Sciences, Otorhinolaryngology, Umeå University, Umeå, 901 89 Sweden; 2 Department of Clinical Sciences, Otorhinolaryngology, Umeå University, Centre for Research and Development, Uppsala University/Region Gävleborg, Gävle, Sweden; 3 Department of Clinical Microbiology, Umeå university, Umeå, Sweden; 4 Umeå Centre for Microbial Research (UCMR), Umeå, Sweden; 5 Department of Medical Biosciences, Umeå University, Umeå, Sweden; 6 Department of Diagnostics and Intervention, Anaesthesiology and Intensive care, Umeå University, Umeå, Sweden; 7 Department of Clinical Sciences, Otorhinolaryngology, Sunderby Research Unit-Norrbotten, Umeå University, Umeå, Sweden

**Keywords:** Subglottic stenosis, Cutibacterium acnes, Whole genome sequencing, Laryngotracheal stenosis

## Abstract

**Purpose:**

Subglottic stenosis (SGS) progressively reduces the airway below the vocal folds. The cause is not known and there is a recurrent need of surgical treatment. Including all phenotypes, SGS affects 1/400 000/yr, with a female dominance. Previous studies have revealed a possible role of the Mycobacterium complex in SGS development. Our hypothesis is that microbiota is associated with the inflammation in SGS, if true it might affect the prevailing treatment options.

**Methods:**

This prospective cross-sectional study included biopsies from 34 patients with subglottic stenosis, collected between 2020 and 2023. Nucleic acids were extracted from the tissue samples and analysed using whole genome sequencing. Microbial composition was characterized using taxonomic profiling of sequencing data. Species with sufficient read counts were selected for further validation using sequence alignment methods to ensure accuracy of identification.

**Results:**

Using the most comprehensive form of genomic testing currently in clinical use, we present curated and stable data on the presence of *Cutibacterium acnes* in 28 out of the 34 cases.

**Conclusion:**

*Cutibacterium acnes* may serve as a driver of the inflammation characterizing SGS and should be considered in therapeutically oriented future studies.

**Level of evidence:**

4.

**Supplementary Information:**

The online version contains supplementary material available at https://doi.org/10.1007/s00405-026-10630-0.

## Introduction

**Fig. 1:**
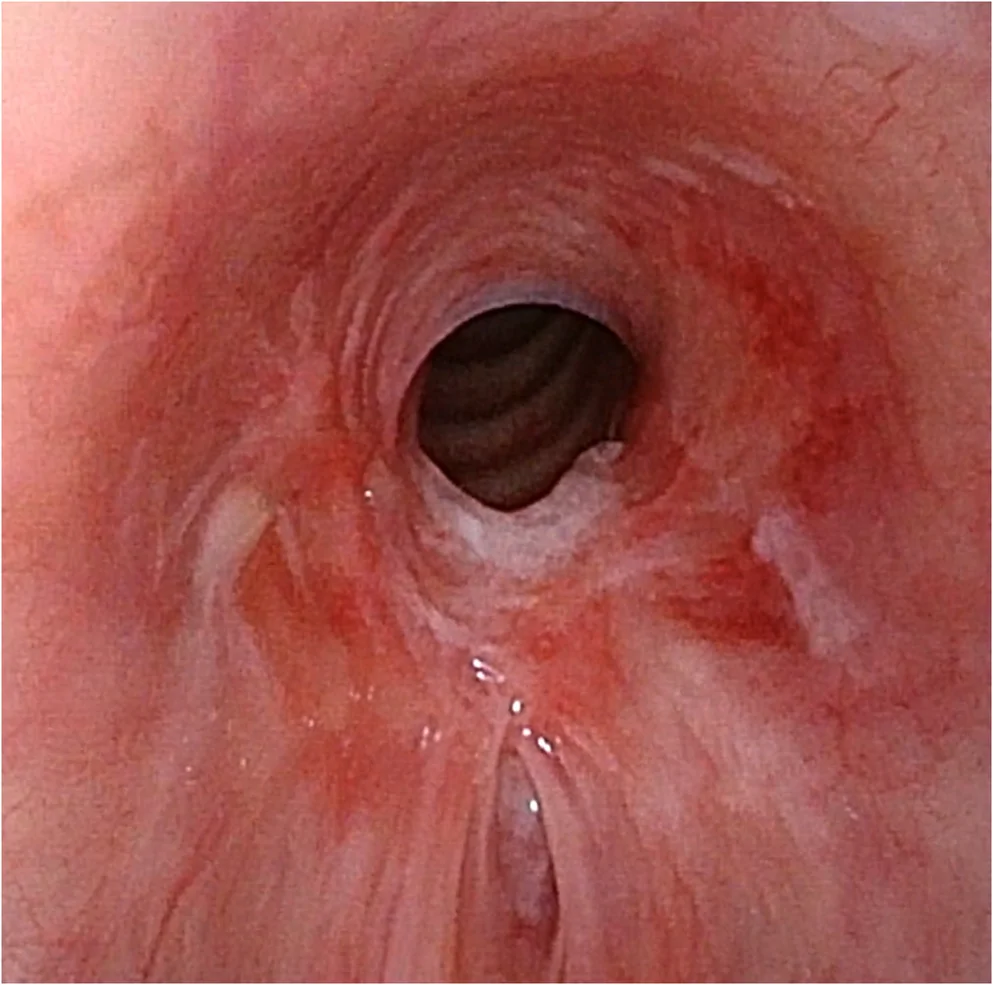
Clinical image of a Subglottic Stenosis. Photo courtesy of prof Katarina Olofsson

Subglottic stenosis (SGS) refers to a narrowing just below the vocal cords with soft tissue histology and unknown cause (Fig. 1). Given the pathogenetic and diagnostic uncertainties involved, reported incidence and prevalence figures are difficult to interpret [1]. Reported incidence data of SGS, as a stratified subtype of laryngotracheal stenosis (LTS) is estimated to 1 of 400,000/yr. This disease is characterized by female dominance, Caucasian origin, progressive loss of airway, huge impact on quality of life as well as a [2] financial healthcare burden comparable to chronic obstructive pulmonary disease (COPD) and diabetes mellitus [3–5]. The unknown cause is the reason for the diversified treatment alternatives with the main goal to keep the airway open. Endoscopic procedures are safe, technically simple, voice-sparing and performed on a day-surgery basis under the prerequisite that the laryngotracheal framework is intact [6]. This technique comprises a combination of radial incision of the scar tissue with CO_2_ laser or cold knife, dilatation of the stenotic part with bougies or ballon and intralesional injection with corticosteroids and/or application of Mitomycin [1]. The use of corticosteroids is motivated by inflammatory scar tissue histology. Scar formation in SGS and skin are reported to be similar and both of unknown origin [7], with an assumption that SGS could be treated in the same way [8]. Time to recurrence shows broad variation; when using endoscopic procedures, 80% of patients are in need of re-operation within 3 years [9]. Open surgical approaches e.g. cricotracheal resection (CTR), by contrast, show evidence of superior long-term results as for airway patency, with approximately 30% risk of recurrence on a 5–10 year basis. However, CTR are associated with morbidity such as anastomotic dehiscence, affected voice quality and need of inpatient hospital care [10]. The recognized pathophysiological features of SGS as well as acne are high androgen levels. In SGS the presence of oestrogen receptors [11] and progesterone receptors [12] is common, and these may interact with TGF-β driven fibroblast performance affecting the inflammation cascade and wound healing. Among other reported upregulated inflammatory pathways in SGS, there is Il-17 A/IL- 23 [13, 14].

Previous studies have reported the presence of microbiota within the intralesional lamina propria, specifically identifying members of the Mycobacterium complex [15]. To try to find possible drivers of the inflammation, we used whole genome sequencing (WGS) describing the metagenome with a comprehensive form of genomic testing currently in clinical use [16].

Since scar formation in SGS and skin are similar, a common driver is possible. *Cutibacterium acnes (C. acnes)*, earlier *Proprionebacterium acnes*, is a commensal part of the human skin flora and a known pathogen in the development of acne vulgaris. *C. acnes* has been reported as a low pathogenic strain with mild local infections but a more potent trigger of the hosts immune response [17]. This capability of *C. acnes* has been implicated as problematic in orthopaedic surgery as wells as silicone breast implants [18]. Stirling A et al. has presented *C. acnes* as a contributor to non-infectious degeneration of lumbar discs [19]. These findings have been reproduced and verified, with an awareness of a possible contamination flora [20, 21].

We hypothesize that a specific microbiota may be necessary, but not solely sufficient, to trigger intralesional inflammation and fibrosis. If confirmed, addressing this knowledge gap could significantly impact therapeutic strategies for SGS and may play a key role in developing individualized, precision medicine-based treatments.

## Materials and methods

### Ethical approval

This study was performed according to the principles of the Declaration of Helsinki and approved by the Swedish Ethical Review Authority reference number; 2020-00253 ratified 2020-04-14 and for the control cases reference number 2021-02941, ratified 2021-12-21. We confirm that all methods were performed in accordance with the relevant guidelines and regulations according to the Strengthening the Reporting of Observational studies in Epidemiology (STROBE) checklist. 

### Study design

Prospective descriptive, cross sectional. 

### Participant recruitment

All patients (new as well as recurrent) with SGS scheduled för endoscopic surgical treatment, between September 2020 and September 2023 were screened for inclusion. SGS was defined as a fibrotic stenosis at the level of the cricoid cartilage verified with awake flexible endoscopy and Computer tomography (CT) scan. Stenosis grading was not included in the study design, as it was not considered essential for addressing the research question. Importantly, these grading systems do not necessarily reflect the underlying biological or tissue-specific characteristics that are central to the present investigation. Exclusion criteria were a malignant, extra thoracal or cartilaginous cause, <18 years of age, multilevel extension, incomplete clinical documentation or not able to provide consent. 

### Surgical settings

All biopsies were obtained during endoscopic balloon dilatation procedure. Patients were assessed under general anaesthesia using rigid suspended laryngoscope and ventilated with superimposed high frequency jet ventilation technique with supraglottic airflow (Twinstream ref: CTNS-110-000). The choice of laryngoscope was guided by the patient's anatomy, ventilation technique and surgeons’ preference’. To reduce risk for contamination we employed systematic sample taking with optimal illumination, and sharp tissue collection using a microscope or airway telescope. The biopsies were approximately 2 x 2 mm and obtained from the stenotic area with sterile laryngeal forceps. The biopsies were placed in a sterile container moistened with saline solutionand promptly transferred to -80 C freezer for later analysis and optimal genetic preservation. No antibiotics were used during the pre-, peri-, or post-operative period if not an infection were detected. 

### Nucleic acid extraction

Subglottic tissue (<20 mg per sample) was stored at -80 C˚. A BeadBlaster™ 24 Microtube homogenizer with steel beads was used to homogenize the tissue (3 × 30 seconds at 4.5 m/s). Viral RNA and DNA and bacterial DNA were extracted by use of a NucleoMag® Pathogen kit (Macherey-Nagel, Germany), following the manufacturer’s instructions, with elution in 43 µl RNase-free water. DNA quantification utilized the Qubit dsDNA HS Assay kit (Thermo Fisher Scientific). To reduce the risk of bacterial contamination, DNA extraction was performed in a safety hood that was decontaminated with UV light before and after the procedure. All steps were carried out using customary protective equipment in a virology laboratory. 

### DNA sequencing and bioinformatics

Sequencing libraries were prepared from the extracted nucleic acids using SMARTer ThruPLEX DNA-seq kit (Takara) and subsequently sequenced on a NovaSeq X Plus. The SMARTer ThruPLEX DNA-seq kit has an optimal requirement of 50 ng DNA per sample. The lower the amount of DNA input, the lower the complexity of the sample. Paired-end 150 bp reads were generated with average 290M read pairs per sample. Taxonomic profiling was performed using KrakenUniq implemented in Taxprofiler v.1.2.4. Classification used the KrakenUniq MicrobialDB 2023-08-08 database. Species identification with >1000 reads were considered for verification. Verification included nucleotide BLAST using the nt database (2025-10-01). 

### Methodology comparative material 

Control biopsies could not be obtained to establish a reliable baseline for comparison. At the same time, it is essential to characterize the laryngeal microbiota without posing any risk of harm to research participants with non-SGS. The comparative material for assessing the laryngeal microbiota is therefore limited to samples collected using, non-invasive methods. Ethical, methodological, and biological constraints prevent the generation of true controls, as conventionally defined:

(i) biopsies from healthy individuals cannot be taken from anatomically relevant laryngeal sites without imposing a substantial risk of permanent tissue damage.
(ii) biopsies from macroscopically healthy larynx in patients with recurrent respiratory papillomatosis (RRP) cannot be collected due to identified risk of iatrogenic HPV transmission to the sampled tissue (iii) postmortem tissue is unsuitable for microbiota analysis, due to degradation of DNA and microbial community composition.

The RRP cases visited the Department of Otorhinolaryngology, University Hospital of Umeå between February 2022 and August 2024. The swabs were collected from macroscopically healthy supraglottic larynx (false vocal cords), using sterile nasopharyngeal swabs (COPAN Cat No 482CE). The RRP cases were anesthetized and ventilated similarly to the SGS cases with the exception that jet ventilation was performed via a catheter under the vocal cords (to reduce risk of Human papilloma virus (HPV) spread in the airway) instead of above the vocal cords as in SGS. The sterile swabs were held by sterile laryngeal forceps and introduced through the rigid laryngoscope. To our knowledge the included swabs tips did not touch any surface other than the healthy tissue on the false vocal folds (supraglottic area). Each swab was put in a Copan tube containing 1 mL Aimes medium and sent by internal post at ambient temperature, or by overnight post from Umeå, to Clinical Microbiology Department in Lund for further analysis.

CultivationFrom each sample, ~10 µL was inoculated onto four agar plates following routine protocols (Clinical Microbiology, Lund, Sweden). A Hem/CNA half‑plate (hematin agar and blood agar with colistin/nalidixic acid plus optochin) and a blood agar plate (PBL‑X) for Streptococcus pneumoniae and β‑hemolytic Streptococcus were incubated in 7% CO₂. A vancomycin‑supplemented plate (URI+V) was used for Gram‑negative selection, and a bacitracin plate (BACI), also incubated in 7% CO₂, for Haemophilus spp. 

### Identification of bacterial species and characterization

Bacterial identification was performed by direct colony MALDI‑TOF MS (Bruker microflex SMART; MBT Compass 4.1; DB‑10833), using ≥2.0 as species‑level score cutoff [22]. Streptococcus dysgalactiae isolates were Lancefield-typed by latex agglutination (Remel). For GBS, five colonies were extracted using MagDES Dx SV 200 on a magLead 12gC systemn, and cps multiplex PCR was performed according to Imperi et al. [23] with minor modifications. Reactions contained Perfecta™ qPCR FastMix™, 0.25 µM primers, and 5 µL template. Thermocycling followed the published protocol, and products were visualized using the QIAxcel system (Qiagen). Serotypes were assigned based on amplicon size relative to reference strains [23]. 

## Results

### Participants

**Fig. 2:**
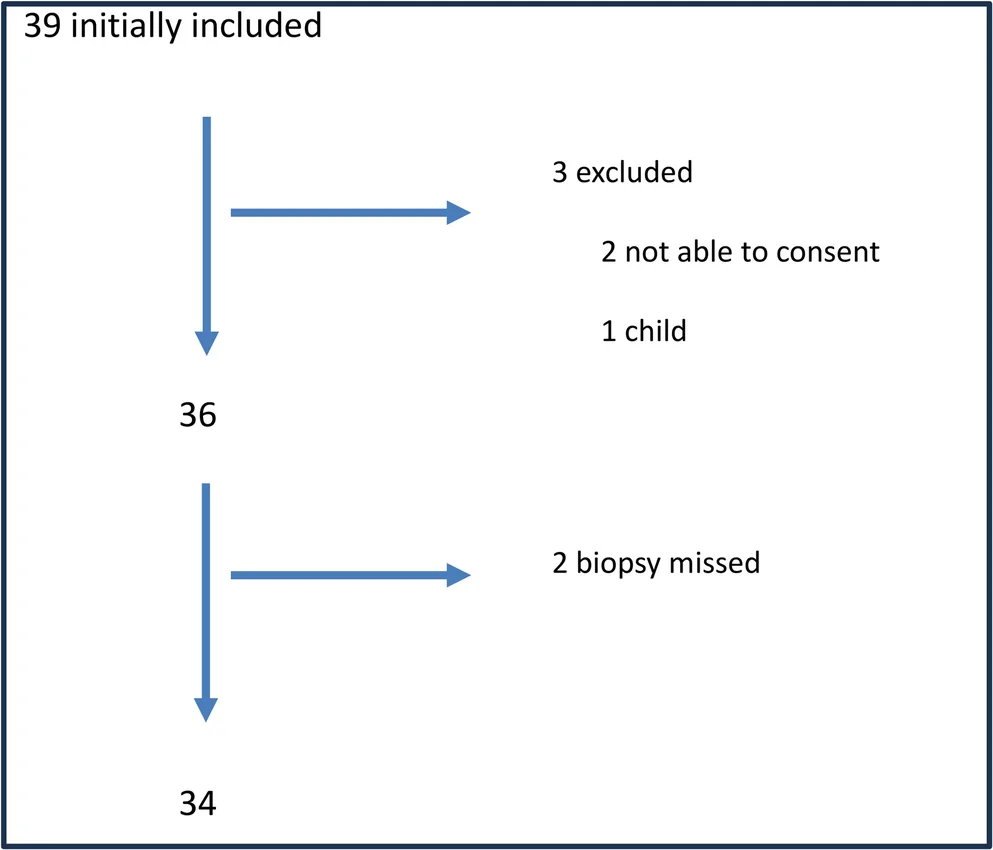
Shows the flowchart for inclusion and exclusion

At baseline, as part of the eligibility assessment, 39 patients were identified. Three were excluded, 2 due to no available written consent and one due to age < 18 yrs. During surgery, biopsies for 2 cases were not performed (Fig. 2). Finally, 34 cases were successfully biopsied and further analysed. The demographics are presented in Table 1. Thirty cases (88.2%, 30/34) were women, and 4 cases (11.8%, 4/34) were men. The mean age was 44.5 years, and the mean BMI was 28. None had an active smoking history. Seven cases (20%, 7/34) had an asthma diagnosis (self-reported and in need of medication) but none had a COPD diagnosis.

**Table 1:** Demographic data of the study population at baseline, in brackets percentage and range

Variable	*n* = 34
Sex	
Male	4 (11.8%)
Female	30 (88.2%)
Age, mean	44.5 (18–79)
BMI, mean	28 (19.9–48.9)
Smoking	
Smoker	0
Non-smoking	20 (58.8%)
No data	14 (41.2%)
Respiratory diseases	
Asthma	7 (20%)
COPD	0

### Whole genome sequencing test results

**Fig. 3:**
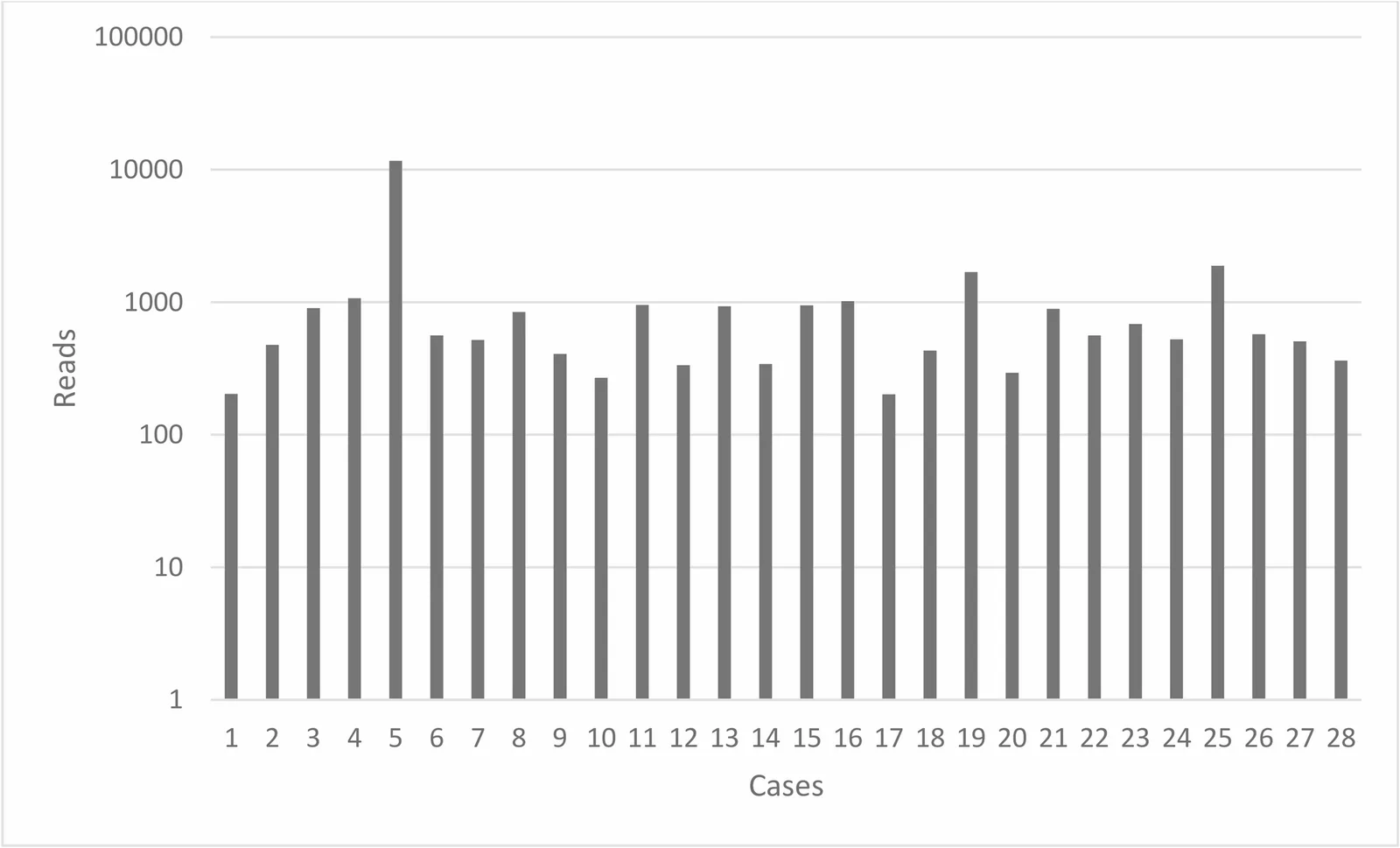
Reads of C. Acnes reported at an individual level in 28 out of 34 cases. Y-axis shown on a log scale

The biopsy sample of each case analysed with WGS technology resulted in approximately 60 identified microbes/individual. The species identification with more than 1000 reads and a following verification with BLAST showed that C. acnes was the most prevalent bacteria detected, present in 28 out of 34 cases. A common finding in cases where classification was verified using BLAST was a high k-mer count, which further supported the accuracy of species identification. The reads and kmers of C. acnes in the 28 cases are shown in (Fig. 3). We also found Roseolovirus humanbeta6b in one case, with a high kmer count, which supports a true finding. Schaalia odontolytica was identified and verified in four cases. Corynebacterium accolens and Micrococcus luteus in one case each. The kmers and reads for the verification of microbes are presented in Table 2.

**Table 2:** Reads and kmers of non-human identified genomes in subglottic stenosis, reported at an individual level in 34 cases and marked with sampleID

SampleID	Species	kmers	Number of reads
ACM66230823	*Cutibacterium acnes*	8061	203
AH67221222	*Micrococcus luteus*	46,572	3347
AH67221222	*Cutibacterium acnes*	21,222	476
AH67221222	*Schaalia odontolytica*	3546	201
AI20230508	*Cutibacterium acnes*	8856	902
AO72201214	*Cutibacterium acnes*	10,062	1072
AR81220509	*Cutibacterium acnes*	57,785	11,669
ASV04210906	*Cutibacterium acnes*	6454	561
BF25220809	*Cutibacterium acnes*	10,814	518
CH48230323	*Cutibacterium acnes*	26,414	843
CR22220516	*Cutibacterium acnes*	4501	407
EA96201022	*Cutibacterium acnes*	7165	269
EJ83210111	*Cutibacterium acnes*	9085	953
IH46230213	*Cutibacterium acnes*	3860	335
IS28220502	*Cutibacterium acnes*	18,465	932
JL01201214	*Cutibacterium acnes*	2470	342
JZ41211105	*Cutibacterium acnes*	6964	948
KE0872109108	*Corynebacterium accolens*	12,918	1278
KE82211115	*Cutibacterium acnes*	12,221	1019
LH61201118	*Schaalia odontolytica*	5707	532
LH61201118	*Cutibacterium acnes*	7124	202
LK40210607	*Cutibacterium acnes*	44,397	432
LL26210614	*Schaalia odontolytica*	20,865	4670
LL26210614	*Cutibacterium acnes*	38,467	1687
LO52201217	*Cutibacterium acnes*	2081	293
LT20220401	*Cutibacterium acnes*	12,058	890
MED44200918	*Roseolovirus humanbeta6b*	99,188	14,718
MED44200918	*Cutibacterium acnes*	21,183	562
MR64211213	*Cutibacterium acnes*	5223	685
NT47230220	*Cutibacterium acnes*	8264	524
SA04210610	*Cutibacterium acnes*	39,026	1882
SG04211130	*Schaalia odontolytica*	2433	611
SG04211130	*Cutibacterium acnes*	7222	573
SH30230803	*Cutibacterium acnes*	6656	507
SS40221025	*Cutibacterium acnes*	12,385	362

### Comparative non- SGS material

Of the 34 non-SGS samples 27 were male and 7 females. The mean age was 48 yrs (20-82). The culture results of the 34 comparative samples revealed the presence of 4 bacterial species: *Haemophilus influenzae, Staphylococcus aureus, Group B Streptococcus (GBS) and Streptococcus equisimilis (Streptococcus dysgalactiae subsp. Equisimilis, SDSE)*. The prevailing species was Staphylococcus Aureus 15 % (5/34), and Hemophilus Influenzae as the second most prevalent 8.9% (3/34). Any of these bacteria was found in 29 % (10/34) of non -SGS cases, as provided in Table 3. 

**Table 3:** Swab controls of the most common bacteria in 20 out of 34 cases

	*n*	%
*Haemophilus influenzae*	3	9
*Staphylococcus aureus*	5	15
*Group B Streptococcus (GBS)*	2	6
Any of *HI, SA* or GBS	10	29

## Discussion

The clinical question in focus for this study is the pathophysiology of SGS and the identification of a possible driver of the confirmed inflammation and fibrotic content of SGS. The implementation of WGS means a very comprehensive form of genomic characterization for clinical use. To reduce the risk of contamination in the tissue sample handling, we performed DNA extraction at a virological (not bacterial) laboratory, with experienced staff and standard protective equipment. We used a nucleic acid extraction method specifically designated for microbial nucleic acid purification to maximize the recovery of non-human DNA. The classification profiling was stabilized against large databases, species with >1000 reads and nucleotid BLAST database were considered for verification of the presence of C. acnes in 28/34 SGS patients. The threshold of 1000 reads are arbitrarily chosen and represents a limitation in metagenomic studies, as there is no clear definition of what constitutes irrelevant reads. 

We employed a brute-force shotgun sequencing approach, in which all DNA is sequenced indiscriminately, rather than using a host-depletion technique to reduce human DNA, as such methods may alter microbial DNA levels in the samples. This approach generates substantially more sequencing data.

One aspect of the tissue handling is that the biopsies were homogenized. As a result, it is not possible to determine whether C. Acnes was located on the surface or deeper within the SGS tissue. Performing microscopy on SGS biopsies could therefore be a relevant next step. 

Several methodological aspects related to sampling strategy and control material warrant careful consideration when interpreting the findings of the present study. Comparative material was obtained using non-invasive swabbing of the supraglottic larynx, whereas SGS samples consisted of subglottic tissue biopsies. Differences in both anatomical location and sampling technique are likely to influence the observed microbial composition. The supraglottic and subglottic regions differ in epithelial characteristics, local oxygen conditions, and airflow dynamics, all of which may affect microbial colonization. In addition, swab-based sampling reflects luminal or mucosal surface microbiota, whereas tissue biopsies may capture bacteria residing within the tissue. Consequently, direct comparison between control swabs and SGS tissue samples must be interpreted with caution. The predominance of *Staphylococcus aureus* in control samples and *Cutibacterium acnes* in SGS tissue may therefore, at least in part, reflect differences in sampling method, anatomical site, and tissue architecture rather than disease-specific microbiota alone. These limitations restrict the ability to fully distinguish between site-related variation, sampling bias, and a true disease-associated microbial signal.

Stratification into idiopathic and non-idiopathic SGS was not undertaken, as the primary objective of the study was to identify microbial characteristics shared across the spectrum of subglottic stenosis rather than to investigate differences between etiological subtypes. Since the cause of SGS has not been established, all phenotypes may be referred to as idiopathic subglottic stenosis (iSGS) or just the holistic term SGS. The variability in reported prevalence and incidence across studies may be explained by differences in how patients are stratified into subgroups based on presumed etiology.

Autoimmune diseases and trauma (e.g., prior intubation or tracheotomy) have been proposed as contributing factors in the development of the stenosis [24]. Some cases are congenital. 

To date, the only proposed causative bacteria or virus previously described is *Mycobacterium* [25]. Previous studies examining the microbiome in SGS have used different sampling methods, where cultivation swabs are most common. Based on our data on the microbial flora in patients (WGS) and controls (swabs), these methods are not optimal in resemblance ratio observations. When comparing stenotic versus non- stenotic laryngeal area in 61 swab samples, *Moraxella* were found to be distinctly associated with the SGS lesion [26]. In a pilot study by Awad et al on 12 swab samples a specific microbiome was suggested defined by *Staphylococcus aureus, Streptococcus parasanguinis, Streptococcus mitis*, and *Corynebacterium pseudogenitalium* in stenosis compared to healthy larynx [27]. In our study, which aimed to compare laryngeal microbiota in SGS larynx with non-SGS larynx without posing any risk, specifically to non SGS participants we employed non‑invasive sampling methods. In accordance to previous findings, *Staphylococcus aureus* emerged as the most detected bacterium in the non-SGS samples (15%). 

Access to anatomically matched subglottic control biopsies was not feasible due to ethical considerations and the risk of iatrogenic injury. Subglottic sampling in non-SGS individuals was considered technically challenging and potentially unsafe in this context. The absence of matched sampling across identical anatomical sites represents an important limitation. Future studies incorporating paired sampling strategies—such as simultaneous swab and tissue sampling within SGS patients—may help differentiate between luminal colonization and tissue-associated microbiota. Tracheal aspirates were considered for this study however they are obtained by suctioning mucus and secretions from the tracheal lumen and are therefore highly influenced by the ventilation strategy employed. In our study, patients were ventilated using Superimposed High-Frequency Jet Ventilation (SIHFJV), a tubeless technique involving continuous humidification and airflow dynamics that differ substantially from conventional ventilation. Consequently, samples obtained through tracheal aspiration (or swabs) would be expected to reflect luminal secretions affected by the ventilation modality rather than accurately represent the microbiological composition of the laryngeal mucosa and therefore deemed not suitable for our study.

 In addition to the frequent detection of the common skin commensal, *Staphylococcus aureus* in non-SGS larynx, we observed that skin-associated bacteria are common also in SGS samples by the presence of *Cutibacterium acnes* (82%, 28/34) C. acnes are enriched in pathways involved in post-operative orthopaedic infections and breast implant surgery with excessive scar formation. The resemblance between excessive scarring in orthopaedic- and breast surgery with SGS histology is striking and unifying by the presence of *C. acnes* as in skin infections [7]. 

In our study it was not possible to rule out whether the *C. Acnes* represented a present or a previous infection. In the present study, *C. acnes* were identified as the most prevalent species, defined as the most frequently detected organism across individuals rather than the most abundant organism within individual samples. It is acknowledged that presence alone does not necessarily reflect biological impact. We acknowledge that the mere presence of a bacterial species does not necessarily reflect its biological impact, and that abundance-related measures may provide additional information regarding its potential role in disease processes. However, there is evidence suggesting that bacterial presence—commonly assessed through microbiome composition or carriage status—can be biologically and clinically relevant in the upper airway and may reflect meaningful host–microbe interactions [28].

Given that effective treatment against *C. Acnes* is currently available, a randomized controlled trial in SGS cases could be considered as a potential next step, with one study arm receiving treatment and the other serving as control.

There is a possibility of contamination with *C. acnes* deposits during intubation procedures affecting the development of SGS. 

Taken together, the consistent detection of *C. acnes* across a majority of SGS tissue samples suggests a potential association with the disease. However, the extent to which this finding reflects a true biological driver, a context-dependent colonizer, or methodological factors cannot be fully resolved within the present study design. The findings should therefore be interpreted as hypothesis-generating and support the need for further studies using refined sampling strategies and improved control material.

## Conclusion

Using WGS, *C. Acnes* was found to be the prevailing microbiota. *C. Acnes* is a known driver of post-operative infections and chronic disease. We speculate that *C. Acnes* may serve as a driver of mucosal inflammation in SGS and as such to be considered in therapeutically oriented future studies.

### Strength and limitations

The prospective design, the clear identification of a knowledge gap, the inclusion of well-defined cases, and the methodological approach constitute key strengths of this study. 

The motivation for using WGS technology was the unknown microbiota in SGS. The obvious challenges using WGS are (i) the generation of large amount of genomic data requiring computational power, (ii) a very high proportion of the data will be from the host genome, (iii) assurance that the classification database contains all relevant species.(iv) Controls are essential to provide a baseline for comparison and preventing bias. In this study, we were unable to fulfil the requirements for controls as a reliable baseline for comparison.  In order to ensure the laryngeal microbiota without any risk of harm to the non-SGS research participant, we chose non-invasive methods 

The microbiota data obtained from non‑SGS and SGS patients constitutes the most methodologically robust and ethically feasible basis for comparative analysis within the study. Although the datasets do not meet the strict scientific criteria for true control groups and were generated using different methodologies, they nevertheless provide a valid representation of the respective microbial communities and thereby fulfill the study’s comparative aims.

Regarding prior interventions as a potential confounding factor. For this study we do not have data regarding previous treatments for SGS and these may influence the local airway environment which could be a confounding factor regarding microbiological findings for this study. 

The observed demographic differences between SGS patients and controls, particularly the predominance of female patients in the SGS cohort and male individuals in the control group, reflect the known epidemiology of the disease but may introduce bias. It remains uncertain whether sex-related biological factors influence the composition of the laryngeal microbiota, and this should be considered when interpreting the findings. 

## Supplementary Information

Supplementary Material 1.

## Data Availability

The data that support the findings of this study are available from the corresponding author SC, upon reasonable request, from other investigators adhering to the European Union General Data Protection Regulation (EU) 2016/679 GDPR.
